# Prevalence and Factors Associated with Peripartum Hysterectomy among Iranian Pregnant Women: A Retrospective Study

**DOI:** 10.4314/ejhs.v32i2.9

**Published:** 2022-03

**Authors:** Saloumeh Peivandi, Sepideh Peivandi, Ali Habibi, Zoleikha Atarod, Mahmood Moosazadeh, Somayeh Fallah

**Affiliations:** 1 Department of Gynecology and Obstetrics, Faculty of Medicine, Mazandaran University of Medical Sciences, Sari, Iran; 2 Student Research Committee, Faculty of Medicine, Mazandaran University of Medical Sciences, Sari, Iran; 3 Gastrointestinal Cancer Research Center, Noncommunicable Diseases Institute, Mazandaran University of Medical Sciences, Sari, Iran

**Keywords:** Postpartum Hemorrhage, Peripartum Period, Hysterectomy, Iran, Pregnant Women

## Abstract

**Background:**

Peripartum hysterectomy (PPH) is one of the effective treatment modalities which is increasingly performed to save the life of pregnant women with uncontrollable severe postpartum hemorrhage. The aim of this study was to assess the prevalence and factors associated with PPH among Iranian pregnant women.

**Methods:**

In a retrospective study, 33 pregnant women with PPH referred to Imam Khomeini Hospital in Sari, Mazandaran province, northern Iran were enrolled. Data were collected using census sampling from March 2017 to 2020. Patients' sociodemographic and clinical characteristics were collected. Fisher's exact test, Kruskal-Wallis, and Mann-Whitney tests were used to evaluate the study variables.

**Results:**

The prevalence of PPH among Iranian pregnant women was 2.81 per 1000 deliveries. The mean length of stay in the hospital and intensive care unit (ICU) was 6.15 (SD=2.91) and 3.17 (SD=1.50) days, respectively. Of the participants, 90.9% had a cesarean section, 51.6% had emergency PPH, 88.2% had emergency PPH in 24 hours after delivery, 9.1% had an induction, and 60.6% had PPH due to placental abnormalities. The mean duration of PPH procedure was 2.51 (SD=1.14) hours. The most common post-operative complication in participants was fever. Participants with older gestational age had more elective PPH (P=0.029). The length of ICU stay was more in patients with total PPH procedure compared to the supracervical (P<0.017). The induction rate was higher in emergency PPH after vaginal delivery compared to cesarean section (P=0.005).

**Conclusion:**

This study showed a high prevalence of PPH among pregnant women. Also, there was a significant relationship between the PPH and length of ICU stay, especially after supracervical hysterectomy. The results of this study can help obstetrician-gynecologist to provide a better intervention for managing patients with postpartum hemorrhage requiring PPH.

## Introduction

Postpartum hemorrhage is the leading cause of maternal deaths worldwide (1). Although over time various medical and surgical interventions have been developed to control or manage postpartum hemorrhage and protect the uterus, nevertheless, it remains a life-threatening condition. Emergency peripartum hysterectomy (PPH) is one of the effective treatment modalities which is increasingly performed to save the life of pregnant women with uncontrollable severe postpartum hemorrhage (2). PPH is used to manage peripheral hemorrhages in labor when other conservative measures to control the hemorrhage and prevent the death of pregnant women are not effective (3). Advanced maternal age, caesarean delivery in previous or current pregnancy, higher parity and abnormal placentation are some of the risk factors for PPH. In addition, giving birth in Asia, poverty, some religious and cultural beliefs, lack of health facilities and poor antenatal care are other important factors for PPH, especially in developing countries (4, 5).

The prevalence of emergency PPH has been reported 0.6 per 1000 deliveries (6). On the other hand, this prevalence reaches less than one person per 1000 deliveries in high-income countries (3, 7, 8). In lower-income countries, the prevalence has been reported between 4 and 11 per 1,000 deliveries (5). The results of a study in Iran revealed that the prevalence of emergency PPH among pregnant women is 1.39 per 1000 deliveries (9). However, the prevalence of emergency PPH appears to increase over time. In the United States, from 1995 through 2007 the prevalence of emergency PPH significantly increased to 15% (10–11); indicating the importance of assessing the prevalence and the factors associated with PPH over time (11). Based on previous limited evidence, PPH is associated with some factors such as abnormal placenta, high number of deliveries and advanced maternal age (7, 8). Also, placental pathology and cesarean section in previous or current pregnancies have shown to be associated with an increased risk of PPH (3, 12). However, these results varied widely across different countries with different demographic, socio-economic and health characteristics of pregnant women (3). Therefore, considering the importance of PPH, as the leading cause of maternal morbidity and mortality, and limited available evidence in Iran, the aim of this study was to evaluate the prevalence and factors associated with PPH among Iranian pregnant women.

## Methods

In a retrospective cross-sectional study, all pregnant women undergoing PPH from March 2017 to 2020 in the department of obstetrics and gynecology at Imam Khomeini Hospital in Sari, Mazandaran province, northern Iran were identified from the medical records. Considering that the prevalence of PPH is relatively low, evaluating its indications and outcomes are often studied retrospectively.

The researchers collected information from 11,741 deliveries (the total number of deliveries including cesarean and vaginal deliveries in the study period) using a researcher-made questionnaire including age, gestational age, the number of babies delivered, gravidity, parity, previous history of cesarean section, history of curettage and abortion, history of placental abnormalities, singleton and multiple pregnancies, the reason for hospitalization, length of stay in hospital and ICU, type of delivery and causes of cesarean section in PPH, type of PPH, type of emergency PPH, induction rate, cause, type and duration of PPH procedure, placental abnormalities, pre and post-operative hemoglobin, needing to blood products, post-operative complications and newborn's condition. In this study PPH was defined as a hysterectomy performed during pregnancy or up to 6 weeks postpartum (5).

**Ethical consideration**: The study was approved by the ethics committee of Mazandaran University of Medical Sciences (code of the research project: 3709). Since this was a retrospective study, patients were not asked to participate in this study. Data was retrieved through medical records after permission and was anonymized to maintain patient confidentiality. Therefore, the ethics committee waived the requirement for informed consent.

**Sample size calculation**: Sample size calculation was based on a low PPH rate of 6% from the previous similar study (3), using a confidence interval of 5% and study power of 95%. An estimated sample of 30 eligible subjects would be needed for this study, so in order to allow for a withdrawal rate of 10%, the investigative team planned to recruit 33 patients.

**Statistical analysis**: Statistical Package for the Social Sciences (SPSS) software package (version 16.0, SPSS Inc., Chicago, IL, USA) was used for data analysis. Normality of the data was evaluated using the Kolmogorov-Smirnov test. Quantitative and qualitative variables were presented via mean (standard deviation) and frequency (percentage). Fishers exact, Kruskal-Wallis, and Mann-Whitney tests were used to evaluate the study variables. Significance was considered less than 0.05.

## Results

A total of 33 patients underwent PPH in the period of three years in our hospital. Age, gestational age, and the number of babies delivered were 31.8 (SD=5.1) years, 35.6 (SD=3.1) weeks, and 1.0 (SD=0.38), respectively. Of pregnant women 60.6% and 45.5% had gravidity and parity 3 and 2, respectively. Also, 39.4% had a history of two cesarean sections, 9.1% had a history of placental abnormalities, and 27.3% had a history of curettage and abortion (Table 1).

**Table 1:** Participants' demographic and clinical characteristics (N=33)

Variable	Participants
**Age (y)**	31.8 (SD=5.1)
**Gestational age (week)**	35.6 (SD=3.1)
**Number of babies delivered**	1.0 (SD=0.38)
**Gravidity**	
1	1 (3.0)
2	10 (30.3)
3	20 (60.6)
4	2 (6.1)
**Pariety**	
1	3 (9.1)
2	15 (45.5)
3	12 (36.4)
4	3 (9.1)
**History of cesarean section**	
1	7 (21.2)
2	13 (39.4)
3	12 (36.4)
4	1 (3.0)
**History of placental** **abnormalities**	
Yes	3 (9.1)
No	30 (90.9)
**History of curettage and** **abortion**	
Yes	9 (27.3)
No	24 (72.7)
**Singleton and multiple** **pregnancies**	
Singleton	31 (93.9)
Multiple	2 (6.1)

The prevalence of PPH among Iranian pregnant women, in this study, was 2.81 per 1000 deliveries. As shown in Table 2, the most common reason for hospitalization of pregnant women with PPH was elective. The mean length of hospital and ICU stay was 6.15 (SD=2.91) and 3.17 (SD=1.50) days, respectively. Of the participants, 90.9% had a cesarean section, 51.6% had emergency PPH, 82.4% had emergency PPH simultaneously or after cesarean delivery, 88.2% had emergency PPH in 24 hours after delivery, 9.1% had an induction, 60.6% had PPH due to placental abnormalities, 54.7% had PPH due to placenta Previa and Accreta, and 84.8% had a procedure of PPH through the total hysterectomy. The mean duration of PPH was 2.51 (SD=1.14) hours. The most common blood products used for the participants were platelets. The most common post-operative complication in participants was fever. 54.6% of infants hospitalized in the neonatal ward.

**Table 2:** Clinical features of participants (N=33)

Variable	Participants
**Reason for hospitalization**	
Labor pain	7 (21.2)
Vaginal bleeding	6 (18.2)
Elective	16 (48.4)
Other	4 (12.2)
**Length of stay (day)**	
Hospital	6.15 (SD=2.91)
ICU	3.17 (SD=1.5)
**Type of delivery in PPH**	
Vaginal	3 (9.1)
Cesarean section	30 (90.9)
**Causes of cesarean section in** **PPH**	
History of cesarean section	22 (66.7)
Fetal distress	5 (15.1)
Multiple pregnancies	1 (3.0)
Placenta Previa	2 (6.1)
**Type of PPH**	
Elective	16 (48.4)
Emergency	17 (51.6)
**Type of emergency PPH**	
After vaginal delivery	3 (17.6)
Simultaneously or after cesarean delivery	14 (82.4)
**Time of emergency PPH**	
24 hours after delivery	15 (88.2)
>24 hours after delivery	2 (11.7)
**Induction**	
Yes	3 (9.1)
No	30 (90.9)
**Causes of PPH**	
Atonic uterus	10 (30.3)
Uterine rupture	2 (6.1)
Metritis	1 (3.0)
Placental abnormalities	20 (60.6)
**Placental abnormalities**	22 (100)
Previa	2 (9.0)
Previa accreta	12 (54.7)
Previa increta	2 (9.0)
Previa percreta	1 (4.6)
Placenta accreta	5 (22.7)
**Procedure of PPH**	
Total	28 (84.8)
Supracervical	5 (15.2)
**Duration of PPH (hour)**	2.51 (SD=1.14)
**Hemoglobin**	
Pre-operative	11.80 (SD=1.08)
Post-operative	10.78 (SD=1.91)
**Blood products**	33 (100)
Fresh frozen plasma	6.04 (SD=3.94)
Platelets	8.06 (SD=5.24)
Platelets concentration	5.06 (SD=3.50)
**Post-operative complications**	
No	16 (48.5)
Bladder damage	5 (15.2)
Intestinal damage	0 (0)
Ureteral injury	0 (0)
Death of the mother	0 (0)
Disseminated intravascular coagulation	0 (0)
Fever	8 (24.2)
Need for re-laparotomy	4 (12.1)
**Newborn's condition**	
Intrauterine fetal demise	1 (3.0)
Hospitalized in the neonatal ward	18 (54.6)
Hospitalized in the NICU	10 (30.3)
No need for hospitalization in the neonatal ward and NICU	4 (12.1)

**PPH**: Peripartum Hysterectomy; SD: Standard Deviation; **ICU**: Intensive Care and mean (standard deviation).

As shown in Table 3, participants with older gestational age had more elective PPH (P=0.029). As shown in Table 4, there was a significant relationship between the procedure of PPH and length of ICU stay (P<0.017). The length of ICU stay was more in the total PPH procedure compared to the supracervical. As presented in Table 5, the induction rate was higher in emergency PPH after vaginal delivery compared to cesarean section (P=0.005).

**Table 3:** Comparison of study variables based on elective and emergency PPH

	PPH		P-value
	
	Elective (N=16)	Emergency(N=17)	
**Age (y)**	32.1 (SD=5.2)	31.5 (SD=5.1)	0.493*
**Gestational age (week)**	36.8 (SD=2.1)	34.4 (SD=3.4)	0.029*
**Gravidity**	2.5 (SD=0.6)	2.8 (SD=0.6)	0.109*
**Pariety**	2.3 (SD=0.7)	2.5 (SD=0.7)	0.321*
**Duration of PPH (hour)**	2.5 (SD=0.8)	2.4 (SD=1.4)	0.448*
**Length of stay (day)**			
Hospital	5.7 (SD=2.0)	6.5 (SD=2.3)	0.263*
ICU	2.6 (SD=0.8)	3.7 (SD=1.9)	0.132*
**Placental abnormalities**			
Previa	2 (15.4)	0 (0)	0.745**
Previa accreta	7 (53.8)	5 (55.6)	
Previa increta	1 (7.7)	1 (11.1)	
Previa percreta	1 (7.7)	0 (0)	
Placenta accreta	2 (15.4)	2 (33.3)	
**Procedure of PPH**			
Total	15 (45.5)	13 (39.4)	0.335**
Supracervical	1 (3.0)	4 (12.1)	
**Blood products**			
Fresh frozen plasma	6.1 (SD=4.4)	6.0 (SD=3.7)	0.875*
Platelets	7.3 (SD=3.5)	8.5 (SD=6.2)	0.510*
Platelets concentration	4.7 (SD=3.5)	5.3 (SD=3.5)	0.587*
**Post-operative complications**			
No	10 (62.5)	6 (35.3)	
Bladder damage	2 (12.5)	3 (17.6)	1**
Intestinal damage	0 (0)	0 (0)	
Ureteral injury	0 (0)	0 (0)	
Death of the mother	0 (0)	0 (0)	
Disseminated intravascular coagulation	0 (0)	0 (0)	
Fever	3 (18.7)	5 (29.4)	0.398**
Need for re-laparotomy	1 (6.3)	3 (17.7)	0.601**

**PPH**: Peripartum Hysterectomy; SD: Standard Deviation; **ICU**: Intensive Care Unit

* p-value was obtained with Mann-Whitney test

** p-value was obtain.ned with Fisher's Exact test

**Table 4:** Comparison of study variables based on total and supracervical PPH

	PPH		P-value
	
	Total (N=28)	Supracervical(N=5)	
**Blood products**			
Fresh frozen plasma	6.0 (SD=3.8)	6.5 (SD=6.3)	0.956*
Platelets	8.0 (SD=5.4)	9.0 (SD=0)	0.480*
Platelets concentration	5.3 (SD=3.4)	3.2 (SD=3.3)	0.136*
**Post-operative complications**			
No	15 (53.5)	2 (40.0)	
Bladder damage	5 (17.9)	0 (0)	0.569**
Intestinal damage	0 (0)	0 (0)	
Ureteral injury	0 (0)	0 (0)	
Death of the mother	0 (0)	0 (0)	
Disseminated intravascular coagulation	0 (0)	0 (0)	
Fever	5 (17.9)	2 (40.0)	0.282**
Need for re-laparotomy	3 (10.7)	1 (20.0)	0.500**
**Length of stay in ICU**	26 (92.9)	2 (7.1)	0.017**

**PPH**: Peripartum Hysterectomy; **SD**: Standard Deviation; **ICU**: Intensive Care Unit

* p-value was obtained with Mann-Whitney test

** p-value was obtained with Fisher's Exact test

**Table 5:** Relationship between induction and duration of PPH based on type of PPH

	PPH			
	
	Elective (N=16)	Emergency (N=17)		P-value
	
		After vaginal delivery	Simultaneously or after cesarean delivery	
**Induction**	0 (0)	2 (66.7)	1 (33.3)	0.005**
**Duration of PPH (hour)**	2.5 (SD=1.0)	1.4 (SD=0.4)	2.9 (SD=1.6)	0.169*

**PPH**: Peripartum Hysterectomy; **SD**: Standard Deviation

* p-value was obtained with Fisher's Exact test

** p-value was obtained with Kruskal Wallis test.

## Discussion

The results of the present study showed that the prevalence of PPH among Iranian pregnant women was 2.81 per 1000 deliveries. Participants with older gestational age had more elective PPH. There was a significant relationship between the procedure of PPH and length of stay in ICU. The length of ICU stay was more in the total compared to the supracervical PPH. The induction rate was higher in emergency PPH after vaginal delivery compared to cesarean section.

Based on the findings of the present study, the prevalence of PPH among Iranian pregnant women was slightly higher than the previous study in Iran, which was reported as 1.39 per 1000 pregnant women (9). Inconsistent with this finding, studies in Nigeria and Pakistan had a prevalence of PPH of 4 and 11 per 1000 pregnant women, respectively (5). However the results of a study in Iraq revealed a lower prevalence of PPH, as 0.4 per 1000 deliveries (13). Also, the prevalence of emergency PPH in Australia and the United Arab Emirates has been reported as 1.1 per 1000 and 0.47 per 1000 deliveries, respectively (14, 15).

Obviously, this difference may be due to the different race/ethnicity and socio-economic situations of participants in different countries. In low-income countries, the prevalence of PPH is usually higher (16). However, the results of a study which was conducted in nine European countries indicate that the prevalence of PPH was vary considerably between high-income countries (17). Additionally, it has been previously shown that race/ethnicity is a risk factor for higher rate of PPH and poor perioperative outcomes and mortality (18).

In the present study, no maternal death occurred. Inconsistent with this finding, a study in India found that perinatal mortality was 62% (19). Regarding maternal mortality, a study in India showed that 14.70% of mothers died (20). In contrast, a systematic review showed that the mortality rate of mothers with emergency PPH was 3% (6). This difference may be due to the higher level of health and treatment programs in prenatal care in recent years and timely treatment in this medical center. Also, delays in timely decisions to undergo PPH surgery can be another possible reason for this difference (21).

The results of the present study indicate a significant relationship between the PPH procedure and length of stay in the ICU. The length of stay in the ICU was more in the total PPH procedure compared to the supracervical. Inconsistent with this finding, a study in Brazil showed that there was no difference between total and supracervical hysterectomy with the length of stay in the ICU (22). This discrepancy may be due to differences in physicians' training, experience, and degrees that lead to different clinical decisions making (23). Another finding of the present study was the higher rate of induction among pregnant women with emergency PPH after vaginal delivery compared to cesarean section. This finding may be due to the increased risk of bleeding after cesarean delivery compared to vaginal delivery. Hence, a study in Norway found that the risk of bleeding at induction of cesarean delivery was 55% higher than vaginal delivery (24).

Overall, the present study showed that the prevalence of PPH among Iranian pregnant women is much higher than in high-income countries (0.6 per 1000 pregnant women) (6). However, further studies are needed to confirm the findings of the present study. There were several limitations in the present study. The most important limitation of this study was the relatively low sample size, which could hamper generalized findings, including the prevalence of PPH. On the other hand, different diagnoses of physicians based on assumptions in their education, experience, and degree can be other limitations.

In conclusion the results of present study revealed that the prevalence of PPH among Iranian pregnant women is 2.81 per 1000 deliveries. Participants with older gestational age had elective PPH. There was a significant relationship between the procedure of PPH and length of stay in ICU. The length of stay in the ICU was more in the total PPH procedure compared to the supracervical. The induction rate was higher in emergency PPH after vaginal delivery compared to cesarean section. This study showed a high prevalence among pregnant women with PPH. The results of this study can help obstetrician-gynecologist to provide a better intervention for managing patients with postpartum hemorrhage requiring PPH. Educating pregnant women for regular antenatal visits and monitored deliveries in the hospital by gynecologists and midwives for timely identification of high risk women and appropriate management is crucial.
